# Pore-Forming Cardiotoxin VVA2 (Volvatoxin A2) Variant I82E/L86K Is an Atypical Duplex-Specific Nuclease

**DOI:** 10.3390/toxins14060392

**Published:** 2022-06-06

**Authors:** Jia-Qi Lu, Jia-Wen Shou, Ka-Ching Lo, Yun-Sang Tang, Wei-Wei Shi, Pang-Chui Shaw

**Affiliations:** 1 Centre for Protein Science and Crystallography, School of Life Sciences, The Chinese University of Hong Kong, Hong Kong, China; lujq@link.cuhk.edu.hk (J.-Q.L.); jiawenshou@cuhk.edu.hk (J.-W.S.); ka_ching_lo@manulife.com.hk (K.-C.L.); samtys@link.cuhk.edu.hk (Y.-S.T.); 2 Li Dak Sum Yip Yio Chin R & D Centre for Chinese Medicine, The Chinese University of Hong Kong, Hong Kong, China; 3 BayRay Innovation Center, Shenzhen Bay Laboratory, Shenzhen 518107, China; shiww@szbl.ac.cn

**Keywords:** VVA2, pore-forming toxin, duplex-specific nuclease, nicking, DNase, metal ion

## Abstract

VVA2 (volvatoxin A chain 2) is a cardiotoxic protein purified from *Volvariella volvacea*. Its biological activities include hemolysis, writhing reaction, neurotoxicity, and ventricular systolic arresting activity. The cytotoxicity of VVA2 was mainly considered due to its pore-forming activity. Here we report a novel biological activity of its variants VVA2 I82E/K86K as a duplex-specific nuclease. Recombinant VVA2 variant I82E/L86K (Re-VVA2 I82E/L86K), deprived of the oligomerization property, shows increased nuclease activity compared to VVA2. Re-VVA2 I82E/L86K converts supercoiled DNA (Replicative form I, RF I) into nicked form (RF II) and linear form (RF III) in the presence of Mg^2+^ or Mn^2+^. Besides plasmid DNA, it also exhibits nuclease activity on *E. coli* genomic DNA rather than ssDNA or RNA. Re-VVA2 I82E/L86K preferentially cleaves dG-dC-rich dsDNA regions and shows the best performance at pH 6–9 and 55 °C. Our structure–function study has revealed amino acid E111 may take an active part in nuclease activity through interacting with metal ions. Based on the sequences of its cleavage sites, a “double-hit” mechanism was thereby proposed. Given that Re-VVA2 I82E/L86K did not exhibit the conserved nuclease structure and sequence, it is considered an atypical duplex-specific nuclease.

## 1. Introduction

A cardiotoxic protein from the edible mushroom *Volvariella volvacea* was previously purified and designated as volvatoxin A (VVA) [1]. It has two components, volvatoxin A1 (VVA1, 44.8 kDa) and volvatoxin A2 (VVA2, 22.4 kDa). VVA was found to have roles in hemolysis, writhing reaction, ventricular systolic arrest and neurotoxicity [2]. The mixture of VVA2 and VVA1 at a weight ratio of 3:1, which is similar to the natural form, shows maximal LD50 toxicity [1]. Interestingly, VVA1 itself was found to inhibit the hemolytic and cytotoxic activities of VVA2. VVA was nontoxic at molar ratios of 2 or lower (VVA2 to VVA1) [3]. Therefore, VVA1 can regulate the activity of VVA2 via direct interaction.

VVA was reported as a heat-labile cardiotoxin by competitively inhibiting Ca^2+^-accumulating activity of sarcoplasmic-reticulum rich microsomal fraction [4]. The Ca^2+^ leakage in the mitochondria suggests there may be a membrane ion channel induced by VVA [4]. The increased Ca^2+^ concentration in the myoplasm was also shown as the reason for VVA causing cardiac arrest in the systole. VVA2 can disrupt the cell membrane in human group O red blood cells, rat liver mitochondria, and cause osmotic swelling of tumor cells [5].

Pore-forming activity of VVA2 was subsequently confirmed [6]. Its N-terminal fragment (NTF, 1–127 residues) is responsible for oligomerization, and its C-terminus (CTF, 166–194 residues) contains a putative heparin-binding site that is responsible for membrane binding and insertion [6,7]. The amphipathic α-helix B of NTF is crucial for VVA2 oligomerization, and the first step that induced the oligomerization was the binding of the toxin to the membrane. The I82E/L86K variant of VVA2 with membrane-binding activity destroyed was reported to lose both hemolytic activity and oligomerization activity in the presence of liposomes [6].

Crystal structures of VVA2 show that its surface is composed of two putative heparin-binding and oligomerization motifs. The electron micrograph of the VVA2 oligomer showed a two-layered helical oligomer model at pH 6.5 [7]. The diameter of the helical oligomer is around 170 Å, with 18 subunits per turn. The thickness of the oligomer is about 55 Å. The helix in the oligomer is left-handed, and the rotation between the dimers is about 40° along the screw axis.

We have purified VVA2 during our initiative originally designated to characterize volvarin, a ribosome-inactivating protein in *Volvariella volvacea* [8]. Further characterization of Re-VVA2 I82E/L86K showed that this protein possesses duplex-specific nuclease activity. Duplex-specific nucleases (DSNs), such as Kamchatka crab-derived DSN and crab DSN, are a group of nucleases specifically targeting dsDNA-containing substrates [9,10,11]. This unusual substrate specificity makes it a promising tool for cDNA library construction, DSN-mediated nucleotide detection/amplification, and RNA sequencing [9,12,13]. This study provides a first-time analysis of this new activity from the VVA2 variant.

## 2. Results

### 2.1. Purification and Characterization of VVA2 from Volvariella volvacea

**Figure 1:**
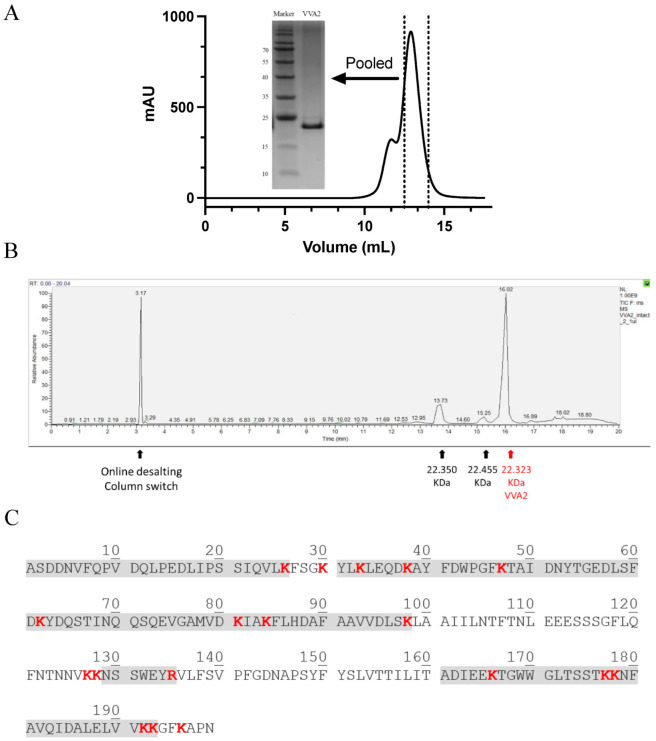
Purification and characterization of VVA2 from mushroom *Volvariella volvacea*. (**A**) Gel filtration profile of purified VVA2 on Sephadex G-75 column. Related fractions were pooled and analyzed with 15% SDS-PAGE gel, stained by Coomassie brilliant blue G-250. (**B**) The purified product in 1A was further checked by Orbitrap LC-MS. The corresponding molecular masses and signals caused by an online desalting column switch are marked. (**C**) Covered peptide sequences from trypsin digested VVA2 were shown in shaded characters. The peptides were matched to the sequence of VVA2 (Accession: 1PP0_A, GI: 52695324). Cleavage sites of trypsin were highlighted in red bold type.

VVA2 was purified from the mushroom *Volvariella volvacea* (Figure 1A), with identity confirmed by Orbitrap LC-MS top-down analysis [1,14]. The protein had a purity of more than 95% and a molecular mass of 22.323 kDa (Figure 1C). The minor peaks were indicated as post-translationally modified forms of VVA2 by Orbitrap bottom-up studies. The trypsin-digested peptides further confirmed the protein was VVA2 (Figure 1B).

### 2.2. VVA2 Has Endonuclease Activity to Convert Circular DNA from Supercoiled form (RF I) to Nicked (RF II) and Linear Form (RF III)

The supercoiled form (RF I) pUC19 plasmid (2686 bp) was incubated with VVA2 or its variant at 37 °C for 60 min in reaction buffer (50 mM Tris-HCl, pH 7.5, 200 mM NaCl) with 10 mM Mg^2+^. Commercial nicking endonuclease Nb. BsrDI (contains only one cut site: GCAATG↓ in pUC19) and restriction enzyme BamH1 were used to indicate nicked (RF II) and linear form (RF III) DNA.

**Figure 2:**
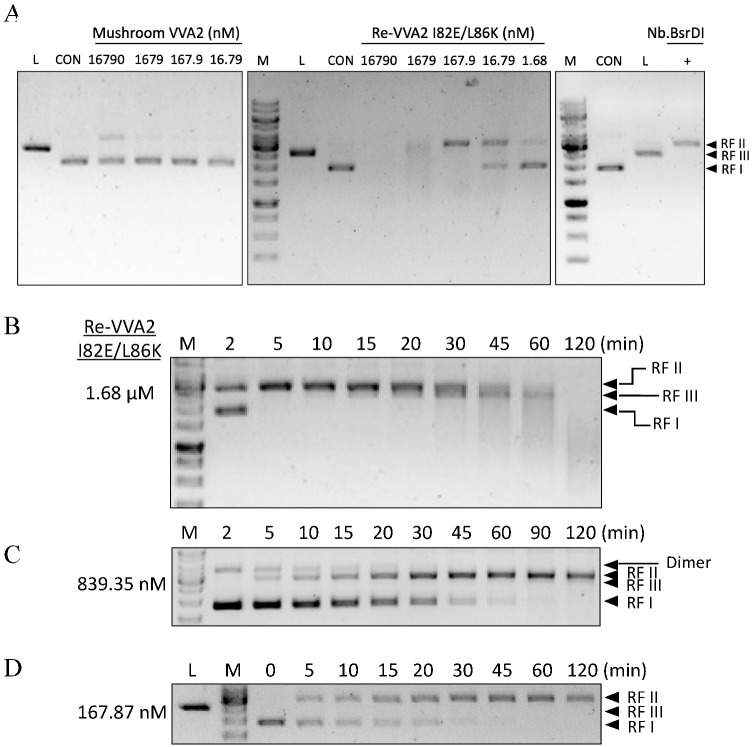
Assay on the endonuclease activity of mushroom VVA2 and recombinant VVA2 I82E/L86K. (**A**) Endonuclease activity of mushroom VVA2 and recombinant VVA2 variant I82E/L86K (Re-VVA2 I82E/L86K). Various amounts of corresponding proteins were incubated with pUC19 (22.26 nM) with 10 mM Mg^2+^ as stated in the Material and Methods. pUC19 treated by Nb. BsrDI and BamH1 were used to indicate nicked and linear DNA (marked as L in the column head). (**B–D**) Endonuclease activity of Re-VVA2 I82E/L86K ((**B**) 1.68 µM, (**C**) 839.35 nM, (**D**) 167.87 nM) on RF I pUC19. Reactions were set as stated in Material and Methods. RF I: replicative form I, supercoiled form; RF II: replicative form II, nicked form; RF III: replicative form III, linear form. M: DNA marker (GeneRuler 1 kb DNA Ladder, Thermo Fisher). CON: control group, pUC19 was incubated without VVA2; L (in the column header): linearized pUC19 by BamH1; reaction products were analyzed on 1% agarose gel.

Wild-type VVA2 extracted from the mushroom (16.8 μM) could only convert ≤ 30% of the plasmid into RF II under Mg^2+^ (Figure 2A). Recombinant VVA2 variant I82E/L86K (Re-VVA2 I82E/L86K) showed more potent activity. DNA was all cleaved while incubated with the same concentration of Re-VVA2 I82E/L86K (16.8 μM). pUC19 digested by 1.68 nM Re-VVA2 I82E/L86K and 16.8 μM mushroom VVA2 showed a similar gel pattern (Figure 2A). The rate of cleavage was in proportion to the concentration of Re-VVA2 I82E/L86K (Figure 2B–D). Re-VVA2 I82E/L86K can act on both supercoiled (RF I) and nicked forms (RF II, by Nb. BsrDI) of pUC19 (Figure S3).

### 2.3. Re-VVA2 I82E/L86K Is a Duplex-Specific Nuclease with a Dependence on Mg^2+^ and Mn^2+^

**Figure 3:**
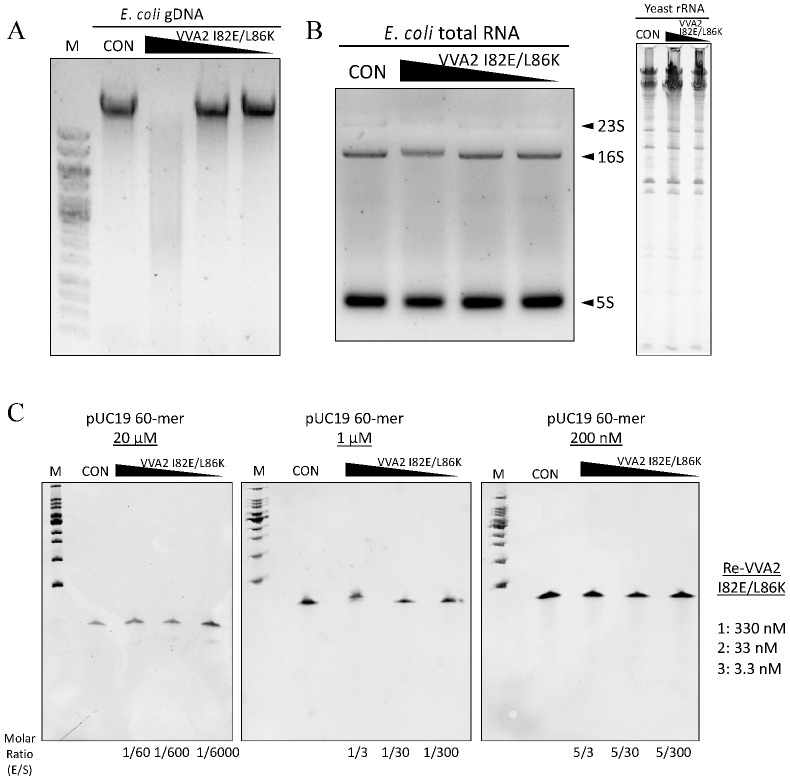
Assay on the nuclease activity of Re-VVA2 I82E/L86K on various nucleic acids. (**A**) Re-VVA2 I82E/L86K on *E. coli* genomic DNA. Re-VVA2 I82E/L86K was used at 833 nM, 83.3 nM, and 8.3 nM for the reaction. CON: control group, *E. coli* genomic DNA incubated with reaction buffer without VVA2. M: DNA marker (GeneRuler 1 kb DNA Ladder, Thermo Fisher, Waltham, NJ, USA). (**B**) Nuclease activity of Re-VVA2 I82E/L86K on *E. coli* RNA and yeast rRNA. Re-VVA2 I82E/L86K at 833 nM, 83.3 nM, and 8.3 nM for *E. coli* RNA was used for the reactions. CON: control group, RNA was incubated with buffer without VVA2. (**C**) Nuclease activity of Re-VVA2 I82E/L86K on 60-mer ssDNA; 200 nM to 20 µM ssDNA and 3.3 nM-330 nM Re-VVA2 I82E/L86K under 20 mM Mg^2+^ were used to assay its nuclease activity. CON: control group, a 60-mer ssDNA incubated with other reagents except for VVA2. M: DNA marker (100 bp DNA Ladder Dye Plus, Takara).

Re-VVA2 I82E/L86K could also cleave *E. coli* gDNA (Figure 3A). However, it did not cleave *E. coli* RNA, yeast ribosomal RNA (rRNA), or an ssDNA 60-mer pUC19 sequence (Figure 3B,C), even at its optimal temperature of 55 °C for 1.5–2 h (Figure S1).

**Figure 4:**
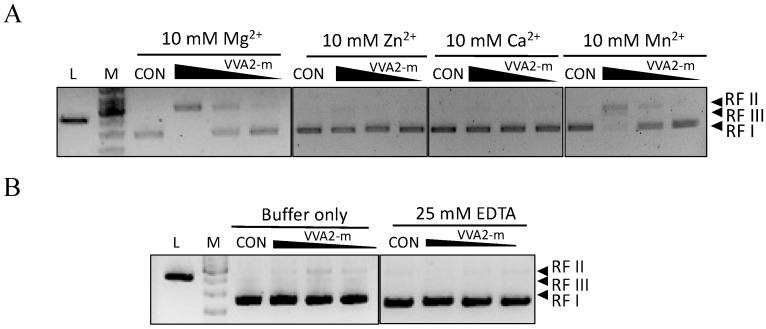
The nuclease activity of Re-VVA2 I82E/L86K is Mg^2+^- and Mn^2+^-dependent. (**A**,**B**) Nuclease activities of Re-VVA2 I82E/L86K under different metal ions (**A**), buffer only, and ethylenediaminetetraacetic acid (EDTA) treated. (**B**) Various concentrations (167.87 nM, 16.79 nM, 1.68 nM) of Re-VVA2 I82E/L86K were used to react under different metal ions as stated in the Materials and Methods. Reaction products were analyzed on 1% agarose gel. VVA2-m: Re-VVA2 I82E/L86K; CON: control group, pUC19 was incubated without VVA2; L (in the column header): linearized pUC19 by BamH1; reaction products were analyzed on 1% agarose gel.

Metal ions Mg^2+^ and Mn^2+^ were found to be essential for the nuclease activity of Re-VVA2 I82E/L86K (Figure 4A,B). Other metal ions such as Zn^2+^ and Ca^2+^ did not mediate the reaction.

### 2.4. Optimal Conditions (Time/pH/Temperature) of VVA2 as an Endonuclease

**Figure 5:**
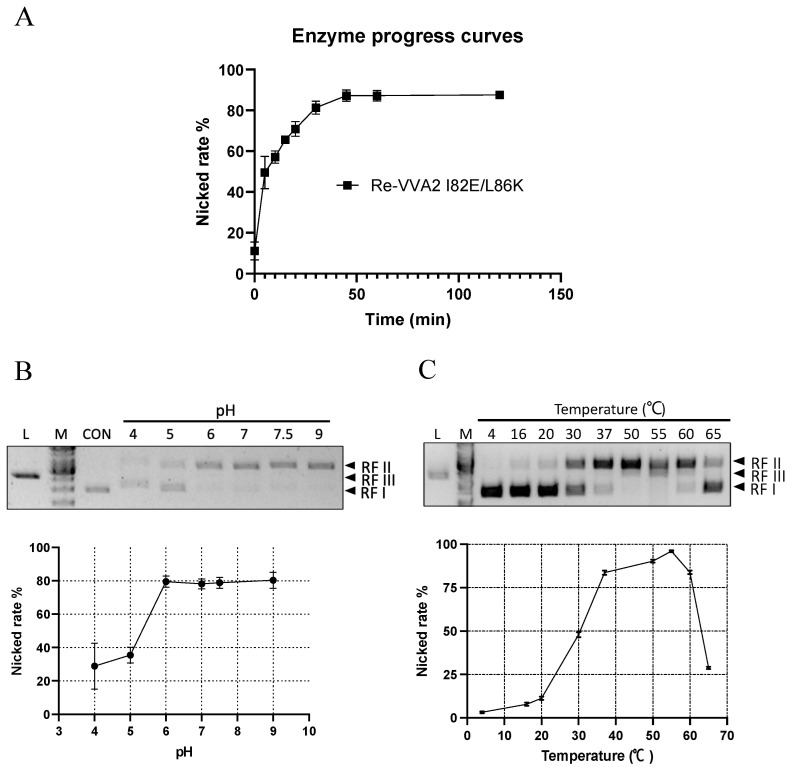
Kinetic characters and optimal conditions (pH/temperature) of Re-VVA2 I82E/L86K as a nuclease. (**A**) The nicking rate of Re-VVA2 I82E/L86K on pUC19 calculated from Figure 2D; 167.87 nM Re-VVA2 I82E/L86K was used. The nicking rates ([RF II]/[RF II + RF I) were calculated and analyzed by ImageJ and GraphPad Prism. N ≥ 3. (**B**) Nuclease activity of Re-VVA2 I82E/L86K at different pH. (**C**) Nuclease activity of Re-VVA2 I82E/L86K at different temperatures. 1% agarose gel and Midori green were used to separate and detect products. Re-VVA2 I82E/L86K concentration: 167.87 nM; RF I–III: replicative form I–III (supercoiled form, nicked form, and linear form DNA); L (in the column header): linearized pUC19 by BamH1; CON: control group, pUC19 incubated with other reagents except for VVA2 variant; M: GeneRuler 1 kb DNA Ladder. Related nicking rates were calculated and analyzed by ImageJ and GraphPad Prism. N ≥ 3. Related data points are shown as average ± SD (standard deviation).

Re-VVA2 I82E/L86K showed time-dependent cleavage of DNA (Figure 5A). At the dosage of 167.87 nM, the nicking reaction occurred mainly in the first 30 min. After 60 min, the amount of total DNA visualized in the gel decreased, suggesting the extensive nicking of the two strands.

The nicking rates ([RF II]/[RF II + RF I]) of Re-VVA2 I82E/L86K were studied at pH 4–9 and temperature 4–65 °C (Figure 5B,C). Re-VVA2 I82E/L86K demonstrated partial cleavage activity at pH 4 and recorded a sharp increase in activity at pH 5, which plateaued at pH 6. No obvious increase in activity was observed when pH further increased to pH 9 (Figure 5B).

Nicking activity of Re-VVA2 I82E/L86K was then tested from 4 °C to 65 °C, with 55 °C as the optimal temperature (Figure 5C). Incubation at 55 °C for 1 h showed a decreased DNA amount and the appearance of RF III DNA. Temperatures at 37 °C to 60 °C all offer nicking rates over 75% (Figure 5C). When temperature was further increased beyond 55 °C, a sharp decrease in activity was observed, likely due to heat denaturation of protein.

### 2.5. VVA2 Cleaves DNA Non-Specifically with Preference in dG-dC-Rich Regions

**Figure 6:**
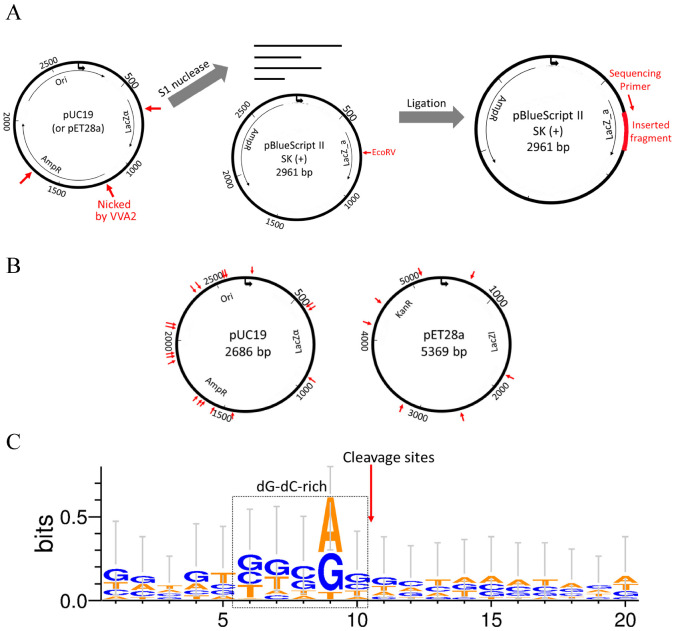
Re-VVA2 I82E/L86K cleaves dsDNA non-specifically with cleavage preference in dG-dC-rich regions. (**A**) The schematic diagram of the experimental design for mapping the cleavage sites of nuclease Re-VVA2 I82E/L86K. The cleaved products (pUC19 and pET28a, mainly in RF II) were digested by S1 nuclease and cloned into pre-linearized pBluescript II SK (+) vector by EcoRV. Colonies were selected and sequenced using universal primer M13F. The detailed protocol is stated in the Materials and Methods. (**B**) Cleavage sites of Re-VVA2 I82E/L86K on pUC19 and pET28a were marked on the related plasmid map. Specific cleavage sites are shown in Table S1. (**C**) Re-VVA2 I82E/L86K has cleavage preference in dG-dC-rich regions. The sequences around cleavage sites were aligned with WebLogo 3. The proposed cleavage sites were marked on the figure.

To investigate the cleavage preference of Re-VVA2 I82E/L86K, cleaved pUC19 and pET28a (mainly in RF II) were incubated with S1 nuclease to remove single-stranded overhangs of the reaction products. Then the fragments with blunt ends were ligated into pBluescript II SK (+) vector, linearized by EcoRV previously (Figure 6A), and recombinant clones were picked for DNA sequencing.

Nine colonies with pUC19 fragments and five colonies with pET28a fragments were sequenced to provide 24 cleavage sites. The listed sequencing results (Table S1) indicated that Re-VVA2 I82E/L86K cleaves DNA non-specifically. The cleavage sites are located in various regions on pUC19 and pET28a (Figure 6B). The sequences around the cleavage sites were dG-dC-rich, suggesting Re-VVA2 I82E/L86K has cleavage preference in dG-dC-rich regions (Figure 6C).

### 2.6. Structure-Function Study of VVA2 Variants

**Figure 7:**
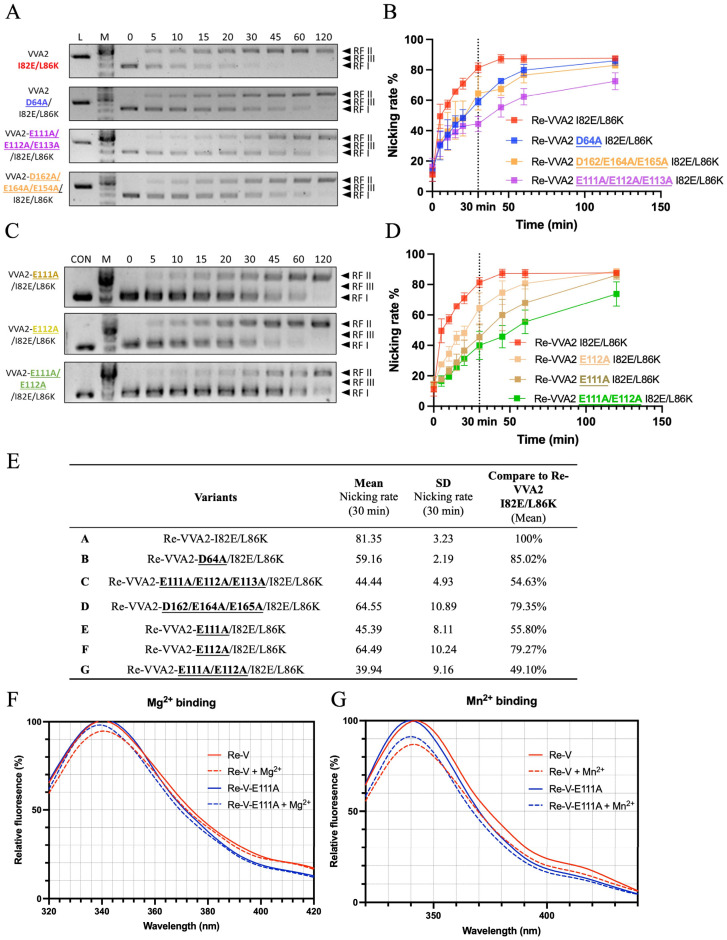
Structure–function relationship and metal ion interaction studies of VVA2 variants. (**A**–**D**) Nuclease activities of VVA2 variants at 0–120 min were studied as stated in the Materials and Methods. Related gel profiles are shown in (**A**) and (**C**), while calculated nicking rates are shown in (**B**) and (**D**). Image J and GraphPad Prism were used to quantify figures and analysis the results. RF I–III: replicative form I–III (supercoiled form, nicked form, and linear form DNA); L (in the column header): linearized pUC19 by BamH1. N ≥ 3. Related data points are shown as average ± SD (standard deviation). (**E**) Nicking rates of VVA2 variants at 30 min. Related mean, SD, and comparison to Re-VVA2 I82E/L86K are shown in the table. (**F**,**G**) Metal ion binding of Re-VVA2 I82E/L86K and its variants. F: Mg^2+^ binding; G: Mn^2+^ binding. Excitation was monitored at 290 nm. Re-V: Re-VVA2 I82E/L86K; Re-V-E111A: Re-VVA2-E111A/I82E/L86K. Data were calculated and analyzed by GraphPad Prism. N ≥ 3.

VVA2 showed low sequence similarity and different active site planning with other DSNs and sugar non-specific nucleases which have a close evolutionary relationship (Figure S2A). To predict how VVA2 interacts with a double-stranded DNA, a 16-mer dsDNA (PDB code: 1CDW) was docked onto VVA2 by SwissDock (Figure S2B). Variants were made to probe the importance of selected amino acids for the nuclease activity (Figure 7). Activities assay showed that E111-113 were important for its nuclease function. The nicking rate of the E111A variant at 30 min decreased to 55.8%, indicating the importance of this amino acid for the enzymatic reaction. This decrease was likely due to the metal ion interaction with the protein, as shown by the tryptophan fluorescence measurement (Figure 7F,G). A decreased signal may be observed in the tryptophan emission spectrum upon association of metal ions, which also indicates the interaction intensity. The result suggested E111 was crucial to substrate DNA cleavage, which may interact with dsDNA via a metal ion.

## 3. Conclusions and Discussion

Volvatoxin A, composed of volvatoxin A1 (VVA1) and volvatoxin A2 (VVA2), are cardiotoxin proteins from the edible mushroom *Volvariella volvacea* [1]. VVA2 was found to be a pore-forming toxin, and the related pre-pore model was proposed [6]. Its functional domains for pore formation have also been identified.

In our study, mushroom VVA2 and Re-VVA2 I82E/L86K was found to have duplex-specific nuclease activity. It can convert supercoiled DNA (RF I) to the nicked (RF II) and linear form (RF III). This is also one of the main characteristics of endonucleases, which could cleave closed circular DNA [15]. Recombinant VVA2 was cloned and expressed to investigate its activity. However, the heterogeneous expression of wild-type VVA2 by *E. coli* was unsuccessful because of its toxicity. Its variant Re-VVA2 I82E/L86K with eliminated oligomerization forming activity was then studied. The nuclease activity of Re-VVA2 I82E/L86K was much higher than the wild-type protein extracted from the mushroom. This increased activity may benefit from the loss of oligomerization, which offers more free active centers for nuclease activity.

The nuclease activity of Re-VVA2 I82E/L86K shows a dose-dependent mode on plasmid DNA. DNA is converted from RF I to RF II within 2 min and then to RF III after 20 min (1.68 µM Re-VVA2 I82E/L86K). A similar phenomenon was observed in nucleases from the mitochondria and vacuole of *N. crassa* [16,17], from barley aleurone layer [18], and from *S. cerevisiae* [19]. They convert DNA from RF I to RF III through RF II, and the latter step can be a slow one [16]. The enzymatic action can be adjusted through enzyme and metal ion concentration [15]. For *N. crassa* mitochondrial nuclease, the 4–8-fold excess enzyme could accelerate the converting of DNA from RF II to RF III in the presence of 10 mM Mg^2+^.

Re-VVA2 I82E/L86K showed substrate specificity. It cleaves double-stranded plasmid and genomic DNA, but not on ssDNA, *E. coli* RNA, or yeast rRNA.

The nuclease activity of Re-VVA2 I82E/L86K is Mg^2+^/Mn^2+^-dependent (Figure 4A). Most nucleases are metal ion dependent. Re-VVA2 I82E/L86K showed high nuclease efficiency in a wide range of pH (pH 6–9) and high temperature (55 °C), which is consistent with most nucleases [15]. However, its molecular weight (22.323 kDa) is relatively lower than that of other DSNs, which are around 42 kDa [9].

The nuclease activity of Re-VVA2 I82E/L86K was considered non-specifically with the preference of dG-dC-rich regions, according to the sequencing results of inserted fragments processed by Re-VVA2 I82E/L86K and S1 nuclease (Figure 6). Similar cleavage preference was observed on Serratia nuclease by Meiss et al. [20]. It cleaves preferentially at dG-dC-rich regions in dsDNA and avoids dA-dT tracts. Endonuclease G from *B. taurus* is also another example [21,22].

Nucleases act on DNA via a “double-hit” or “single-hit” mechanism. The former indicates nucleases that nick at different sites on each strand randomly. DNA cleavage is achieved when the two strands are nicked at the same position [15]. The latter mechanism is for those which nicks at the same site on both strands in a single hit [23]. According to our study on the cleavage sites and action mode, Re-VVA2 I82E/L86K was considered using the “Double-hit” mode.

The multiple sequence alignment showed that VVA2 shares low similarity with other DSNs such as duplex-specific nuclease from *Paralithodes camtschaticus* and its evolution neighbor non-specific nucleases (Figure S2A). The active centers of NucA, NucB, and mitochondrial EndoG have a conserved “ββα-metal” structure [24,25]. However, in VVA2, only a ββα-like structure with a different orientation of the β-strands was found. This suggests VVA2 may be an atypical non-specific nuclease with a different mechanism.

The possible interaction of negatively charged DNA can occur as (1) the direct interaction with positively charged amino acid (aa); (2) the interaction with aa main chain (N and O); and (3) the interaction with negatively charged aa through the mediation of metal ion Mg^2+^ or Mn^2+^. To investigate the structure–function relationship of VVA2 variants, amino acids that may interact with DNA through Mg^2+^ or Mn^2+^ were studied with a priori regarding the indispensable role of metal ions in its nuclease activity. Molecular docking was conducted to narrow the scope (Figure S2B). Amino acids located around with negative charge were selected for mutagenesis study, including additional mutation besides I82E/L86K on D64, E111–113, and D162/E164/E165. The nuclease activities of these variants show that E111–113 may be essential to its activity, and E111 was the most significant one, which affects the nuclease activity through metal ion interaction (Figure 7E–G).

**Figure 8:**
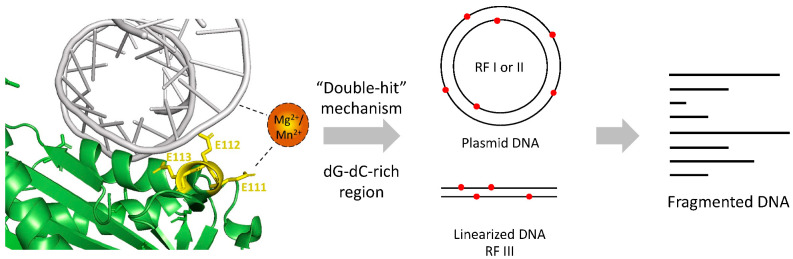
Proposed mechanism of Re-VVA2 I82E/L86K as a duplex-specific nuclease (DSN). Re-VVA2 I82E/L86K interacts with DNA with the mediation of Mg^2+^ or Mn^2+^. E111 plays an important role in the interaction. The “double-hit” mechanism may be used in the cleavage reaction on plasmid DNA and linearized DNA, such as genomic DNA, finally resulting in fragmented DNA.

A proposed mechanism of Re-VVA2 I82E/L86K was made based on our study (Figure 8). With the help of E111, Re-VVA2 I82E/L86K interacts with duplex DNA mediated by metal ion Mg^2+^ or Mn^2+^. Re-VVA2 I82E/L86K nicks at various sites at both strands using a “double-hit” mechanism. When the random cleavage encounters the opposite position at both strands, it finally results in the complete scission of the duplex DNA. Benefiting from its non-specific cleavage and duplex-specific characters as a DSN, Re-VVA2 I82E/L86K is a promising tool for cDNA library construction [26], circulating miRNA detection [27], and single nucleotide polymorphism (SNP) recognition [28].

## 4. Materials and Methods

### 4.1. Materials

*Chemicals and Reagents:* Acetic acid glacial (CH_3_COOH, 99.0%, Ducsan, Republic of Korea); Ammonium sulfate (USB Corporation, Cleveland, OH, USA); Phenylmethanesulfonylfluoride (PMSF, VWR International, Radnor, PA, USA); Sodium acetate (Sigma-Aldrich, St Louis, MO, USA); Sodium chloride (ChemCruz, Dallas, TX, USA); TopVision Agarose (Thermo Fisher Scientific, Waltham, MA, USA); DNA Gel Loading Dye (Thermo Fisher Scientific, Waltham, MA, USA); Midori Green Advance (Nippon Genetics, Düren, Nordrhein-Westfalen, Germany); 100 bp DNA Ladder Dye Plus (Takara, San Jose, CA, USA); 1 Kb Plus DNA Ladder (Thermo Fisher Scientific, Waltham, MA, USA); 60-mer ssDNA from pUC19: 5′- TGACA CCACG ATGCC TGTAG CAATG GCAAC AACGT TGCGC AAACT ATTAA CTGGC GAACT-3′.

*Kits:* GeneJET PCR Purification Kit (Thermo Fisher Scientific, Waltham, MA, USA); GeneJET Gel Extraction Kit (Thermo Fisher Scientific, Waltham, MA, USA); Pierce Peptide Desalting Spin Columns (Thermo Fisher Scientific, Waltham, MA, USA).

*Cells:* DH5α (Thermo Fisher Scientific, Waltham, MA, USA); OverExpress C43(DE3) cell (Cat. No. CMC0019, Sigma, St Louis, MO, USA); LB Broth Base (Thermo Fisher Scientific, Waltham, MA, USA); Isopropyl β-d-1-thiogalactopyranoside (IPTG, Sigma-Aldrich, St Louis, MO, USA). Plasmid: pET28a, pUC19, pBluescript II SK (+) vector (Addgene, Watertown, MA, USA). Enzyme: Phusion High-Fidelity DNA Polymerase (New England Biolabs, NEB, Ipswich, MA, USA); BamHI (NEB); T4 DNA ligase (NEB); S1 nuclease (Thermo Fisher Scientific, Waltham, MA, USA); EcoRV (NEB).

*Equipment:* HiTrap CM FF column (Cytiva, Marlborough, MA, USA); Gel Doc EZ System (Bio-Rad, Hercules, CA, USA); High-Speed Centrifuge Beckman Avanti JXN-30 (Beckman Coulter, Indianapolis, IN, USA); Rotor JA-20 (Beckman Coulter, Indianapolis, IN, USA) (≥ 30,000× *g*); Rotor JA-14 (Beckman Coulter, Indianapolis, IN, USA) (≥12,000× *g*); Sephadex G-75 column (Cytiva, Marlborough, MA, USA); Orbitrap Fusion Lumos Tribrid Mass Spectrometer (Thermo Fisher ScientificOrbitrap Exploris MX Mass Detector (Thermo Fisher Scientific, Waltham, MA, USA); ThermoMixer (Eppendorf, Hamburg, Germany); Ni-NTA Agarose (Bio-Rad, Hercules, CA, USA); Imidazole (Sigma-Aldrich, St Louis, MO, USA); Eppendorf BioSpectrometer basic (Eppendorf, Hamburg, Germany); thermal cyclers (Applied Biosystems, Waltham, MA, USA); flow cell disrupter JN-Mini (JNBIO, Guangdong, China); AKTA Prime (Cytiva, Marlborough, MA, USA); with Superdex 75 10/300 GL gel filtration column (Cytiva, Marlborough, MA, USA); Tecan Spark 10 M Microplate Reader (Tecan, Männedorf, Switzerland).

*Software:* Image J (Version 1.53, National Institute of Mental Health, NIH, Bethesda, MD, USA); GraphPad Prism (Version 9.3.1, GraphPad Software, San Diego, CA, USA); GenSmart Codon Optimization tool (GenScript, Piscataway, NJ, USA); Thermo Scientific Xcalibur (Thermo Fisher Scientific, Waltham, MA, USA); Image Lab Software (Bio-Rad, Hercules, CA, USA); PyMol (DeLano Scientific LLC, Palo Alto, CA, USA).

### 4.2. Protein Purification of Volvatoxin A2 (VVA2) from Volvariella volvacea

VVA2 was purified from the mushroom *Volvariella volvacea* based on the previous studies [1,14]. Mushrooms were bought from the local market in Hong Kong. After grinding and extracting with 0.05 M acetic acid at 4 °C overnight, total proteins were precipitated with 95% saturation of (NH_4_)_2_SO_4_. The precipitated proteins were obtained by centrifugation at 11,655× *g* (Rotor JA-14) for 30 min. The pellet was collected and dissolved in 10 mM sodium phosphate, pH 7.2, and dialysis against the same buffer. Impurities were separated by gel filtration with Sephadex G-75 column in the same buffer. Buffer exchange with 10 mM sodium acetate, pH 3.5, was conducted before the HiTrap CM FF column for better binding. HiTrap CM FF column coupled with the DEAE column was used for final purification. An unabsorbed HiTrap CM FF column fraction was loaded on the DEAE column and eluted with 0–0.3 M NaCl. The major peak’s protein was collected and further separated with the Sephadex G-75 column. The peaks containing purified VVA2 were collected and confirmed with SDS-PAGE. The concentration of related protein was determined by spectrometers and the BCA method [29]. Extinction coefficient: 31,720 M^−1^ cm^−1^.

### 4.3. Orbitrap MS Analysis of Intact VVA2 and Trypsin-Digested VVA2 Fragments

According to the manufacturer’s instruction, to analyze intact protein by Orbitrap MS, purified wild-type VVA2 protein solution was desalted by Pierce peptide desalting spin columns. For identification, liquid chromatography (LC) was performed on column Thermo Scientific PepMap 300 C4 HPLC (online desalting) column followed by Acclaim 300 C18 HPLC column as stated in the previous study [30]. Thermo Scientific Xcalibur software was used to analyze signals detected by Orbitrap Exploris MX Mass Detector.

To analyze trypsin digested protein, a clean needle was used to ground SDS-PAGE gel bands. More than three times, the gel bands were distained with 200 µL 50% MeOH/10 mM NH_4_HCO_3_. The distained gel was dehydrated by acetonitrile (ACN) and digested in 20 ng/µL trypsin digestion for 4 °C overnight. Then, 10 min sonication was conducted to extract the digested products with 5 µL 80% acetonitrile/2.5% TFA. After that, 1 μL purified products were injected into the Orbitrap Fusion Lumos Tribrid Mass Spectrometer before being desalted by the same spin columns, as stated previously [30].

### 4.4. Cloning and Site-Directed Mutagenesis of VVA2

The DNA sequence of VVA2-I82E/L86K was optimized by the GenSmart Codon Optimization tool. The related DNA fragment was synthesized by GenScript (Piscataway, NJ, USA) and cloned to pET28a. Overlapping PCR was used for site-directed mutagenesis as previously reported [31]. Primers used in site-directed mutagenesis are listed (Table S2). The ligation products were transformed into DH5α, spread on LB agarose plates, and sequenced by BGI (Beijing Genomics Institute, Shenzhen, China).

### 4.5. Heterogeneous Expression and Purification of Recombinant VVA2 Variants

OverExpress C43(DE3) cells were used to express recombinant VVA2 variants. The overnight cultured starter was added into the fresh, sterilized LB broth medium at 1/100 (*v*/*v*). The shaker incubator at 220 rpm 37 °C made the inoculated medium growth to OD 0.8–1.0 after 3.5 h. Then, 0.1 mM IPTG was added to induce the protein expression at 25 °C overnight. Cells were harvested by centrifugation at 11,655× *g* for 4 min. Flow cell disrupter JN-Mini pulverized the *E. coli* cells at 1200 bar and 4 °C in buffer A (20 mM Tris pH 7.5, 100 mM NaCl, 50 mM Imidazole, 5% glycerol) with 1mM PMSF (Phenylmethanesulfonylfluoride). Centrifugation at 31,360× *g* (Rotor JA-20) was conducted to remove cell debris. Pre-equilibrated Ni NTA beads by buffer A were used for further purification. After loading the cell lysate twice on the column, it was washed by buffer A for 10 column volumes (CV), and then by buffer B (20 mM Tris pH 7.5, 100 mM NaCl, 75 mM Imidazole, 5% glycerol) for another 10 CV. The purified VVA2 variants were eluted with buffer C (20 mM Tris pH 7.5, 100 mM NaCl, 300 mM Imidazole, 5% glycerol). The final products were purified by AKTA Prime with the Superdex 75 10/300 GL gel filtration column. The yield was about 50 µg of protein per liter of cells in LB medium.

### 4.6. Nuclease Activity Assays of VVA2 Variants

*Action on plasmid DNA:* a 10 µL reaction system was used in the nuclease activity assays of VVA2 variants unless there was a special indication. Supercoiled DNA pUC19 (22.26 nM) was incubated with 167.87 nM VVA2 (or varied to 45–3600 nM according to specific usage) at 37 °C for 60 min in 50 mM Tris pH 7.5, 200 mM NaCl. Metal ions were added to a final concentration of 10 mM. The reaction temperatures were controlled by a ThermoMixer and thermal cyclers according to the reaction volume. Then, 1 M sodium acetate at pH 4, 5, 6, and 1M Tris-HCl at pH 7, 7.5, and 9 were used to adjust the reaction pH as needed. The phenol/chloroform method [32] or the GeneJET PCR Purification Kit were used to stop the reaction and extract the DNA. Samples were applied on 1% agarose gel with standard TBE buffer and visualized by the Gel Doc EZ system. Midori Green Advance was used to destain corresponding gel bands.

*Action on E. coli genomic DNA: E. coli* genomic DNA (gDNA) was extracted as previously stated [33]. The concentration of the genomic DNA was adjusted to 2 mg/mL with nuclease-free water. Then, 30 µL reaction mixture was prepared as 2 µL (43 pM) gDNA, with an indicated amount (833 nM, 83.3 nM, 8.3 nM) of Re-VVA2 I82E/L86K in reaction buffer (50 mM Tris pH 7.5, 200 mM NaCl, nuclease-free). Mg^2+^ was added to the final concentration of 20 mM. The reactions were incubated at 37 °C for 60 min. After that, 0.5% agarose gel was used to analyze the final products.

*Action on RNA: Saccharomyces cerevisiae* yeast ribosomes and *E. coli* total RNA were extracted as previously stated [34,35]. Then, 30 µL reaction was set as 500 ng RNA, with an indicated amount (833 nM, 83.3 nM, 8.3 nM-for *E. coli* RNA) of Re-VVA2 I82E/L86K in reaction buffer (50 mM Tris pH 7.5, 200 mM NaCl, nuclease-free). Mg^2+^ was added to the final concentration of 20 mM. The reaction was incubated at 37 °C for 60 min. The reaction mixture was precipitated with 75% ethanol twice and analyzed with 1% agarose gel (total RNA) and 8 M Urea/6% acrylamide gel (rRNA), stained with Midori Green Advance and EtBr, respectively.

*Action on ssDNA:* 60-mer ssDNA was synthesized by Life Technologies (Waltham, MA, USA). The sequence mimics a fragment from pUC19. Then, 200 nM to 20 µM ssDNA was incubated with 3.3–330 nM Re-VVA2 I82E/L86K under 20 mM Mg^2+^ at 37 °C for 2 h as indicated in the related figures in reaction buffer (50 mM Tris pH 7.5, 200 mM NaCl, nuclease-free). The reaction mixture was precipitated with 75% ethanol twice, and analyzed with 8 M Urea/15% acrylamide gel, stained with EtBr.

### 4.7. Mapping of Cleavage Sites of VVA2 Variants

To map the cleavage sites of VVA2 variants, Re-VVA2 I82E/L86K was added to the reactions to convert pUC19 and pET28a from RF I to RF II (main product) and RF III. S1 nuclease was used to cleave the single-strand overhang of resulting RF II/III DNA into blunt ends, according to the manufacturer’s instruction. Moreover, the fragments were purified by the Gel Extraction Kit. The pre-linearized pBluescript II SK (+) vector by EcoRV was ligated with the purified fragments, transformed to DH5α competent cells, and screened by blue-white screening [36]. Positive colonies were incubated in LB medium overnight and sent to sequencing by BGI using universal primer M13F. Sequence analysis of the fragments was conducted by NCBI blastn program (access date: 16 February 2022) with sequences of pUC19/pET28a and pBluescript II SK (+) vectors from Addgene (Watertown, MA, USA).

### 4.8. Sequence Analysis and Docking of the DNA to VVA2

Multiple sequence alignment was conducted with Blosum62 [37] and presented with ENDscript2 [38]. The sequences around cleavage sites were analyzed using WebLogo 3 (http://weblogo.threeplusone.com/create.cgi, accessed on 30 March 2022). A randomly selected 16-mer DNA (PDB code: 1CDW) was docked on VVA2 (PDB code: 1PP0_A) by SwissDock, as previously reported [39]. The docking results were presented by PyMol. The negatively charged amino acids that may interact with the DNA 16-mer were marked and selected for the subsequent mutagenesis studies.

### 4.9. Agarose Gel Quantification

The agarose gel was visualized and imaged by the Gel Doc EZ system. Image Lab Software was used to adjust and export figures. Subsequently, quantification was conducted by Image J. Corresponding nicked and supercoiled form DNA were indicated by Nb. BsrDI and BamHI cleaved pUC19. The nicking rates were calculated as this formula: [RF II]/[RF II + RF I]. GraphPad Prism was used to calculate the mean and SD (standard deviation), and to visualize the data.

### 4.10. Metal Ion Binding Assay

The metal ion binding capacity of VVA2 variants was conducted based on intrinsic tryptophan fluorescence (ITF) measurements [40,41], as previously reported. Recombinant VVA2 variants without free metal ions (pre-treated with EDTA and buffer exchange, EDTA: Ethylenediaminetetraacetic acid) were diluted to 10 µM in assay buffer (50 mM Tris-HCl, pH 7.5, 100 mM NaCl). Metal ions (Mg^2+^ or Mn^2+^) were added to the final concentration of 20 mM. A Tecan Spark 10M microplate reader was used to measure the intrinsic fluorescence. Excitation was set as 290 nm, while emission scan was set as 310–440 nm. The bandwidth was adjusted to 5 nm, with a step size of 2 nm. Background fluorescence was deducted using the buffer with a similar operation. Spectra with more than three repeats were analyzed and presented with GraphPad Prism.

## Data Availability

All data is contained within this paper.

## Supplementary Materials

The following are available online at https://www.mdpi.com/article/10.3390/toxins14060392/s1. Figure S1. The nuclease activity of VVA2 variants on ssDNA and *E. coli* total RNA under optimal conditions for a longer time; Figure S2. The multiple sequence alignment and structural comparison of VVA2 and other members of sugar non-specific nuclease; Figure S3. The comparison of nuclease activity of Re-VVA2 I82E/L86K on supercoiled and nicked dsDNA; Table S1. The cleavage sites of Re-VVA2 I82E/L86K on pUC19 and pET28a; Table S2. The primers used for cloning and site-direct mutagenesis1 studies of VVA2.
