# Supplementary material for: Pore-Forming Cardiotoxin VVA2 (Volvatoxin A2) Variant I82E/L86K Is an Atypical Duplex-Specific Nuclease
Source: Toxins (Basel). 2022 Jun 6;14(6):392. doi: 10.3390/toxins14060392 (PMC9230820; doi:10.3390/toxins14060392)
Supplement: Supplementary file 1 [file toxins-14-00392-s001.zip › toxins-1743836 - Supplementary Material.pdf]

Supplementary Materials: Pore-Forming Cardiotoxin VVA2 (Volvatoxin A2) Variant I82E/L86K is an Atypical Duplex-Specific Nuclease

Jia-Qi Lu, Jia-Wen Shou, Ka-Ching Lo, Yun-Sang Tang, Wei-Wei Shi and Pang-Chui Shaw

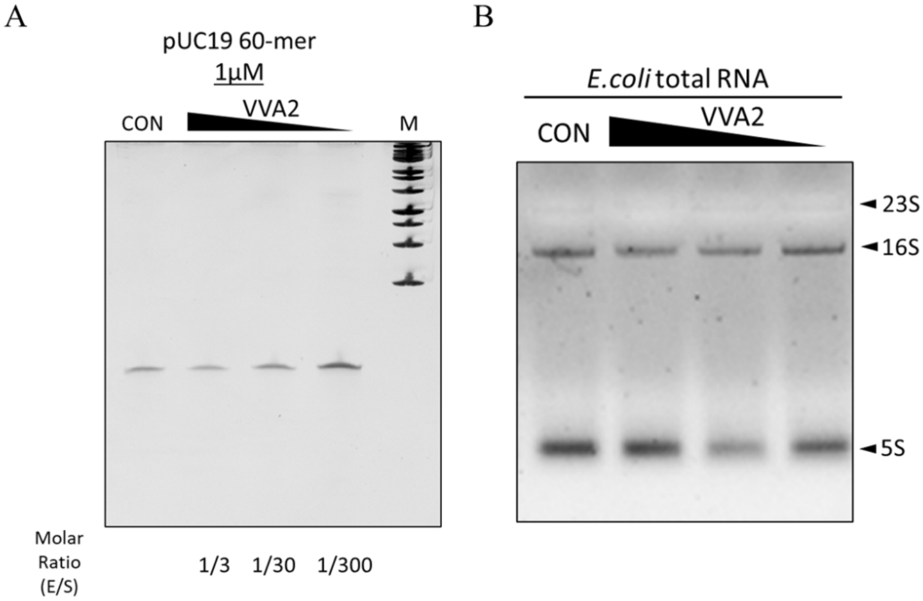

Figure S1. The nuclease activity of VVA2 variant on ssDNA and *E. coli* total RNA under optimal conditions. (**A**) Nuclease activity of Re-VVA2 I82E/L86K on 60-mer ssDNA mimics a fragment from pUC19. 1 μM ssDNA and 3.3 nM/33 nM/330 nM Re-VVA2-I82E/L86K under 25 mM Mg^{2+} were used to assay its nuclease activity at 55°C for 2 h in reaction buffer (50 mM Tris pH 7.5, 200 mM NaCl, nuclease-free) as stated in Material and Methods. 8 M Urea/ 15% acrylamide gel stained with EtBr was used to analyze the product. CON: control group, ssDNA 60-mer incubated with other reagents except VVA2. M: DNA marker (100 bp DNA Ladder Dye Plus, Takara). (**B**) Nuclease activity of Re-VVA2 I82E/L86K on E. coli total RNA and yeast rRNA. Series diluted Re-VVA2-I82E/L86K (833 nM, 83.3 nM, and 8.3 nM) in reaction buffer (50 mM Tris pH 7.5, 200 mM NaCl, nuclease-free) was used under 25 mM Mg^{2+} at 55°C for 1.5 h. 1% agarose gel was used to analyze the products after 75% ethanol precipitation. CON: The control group.

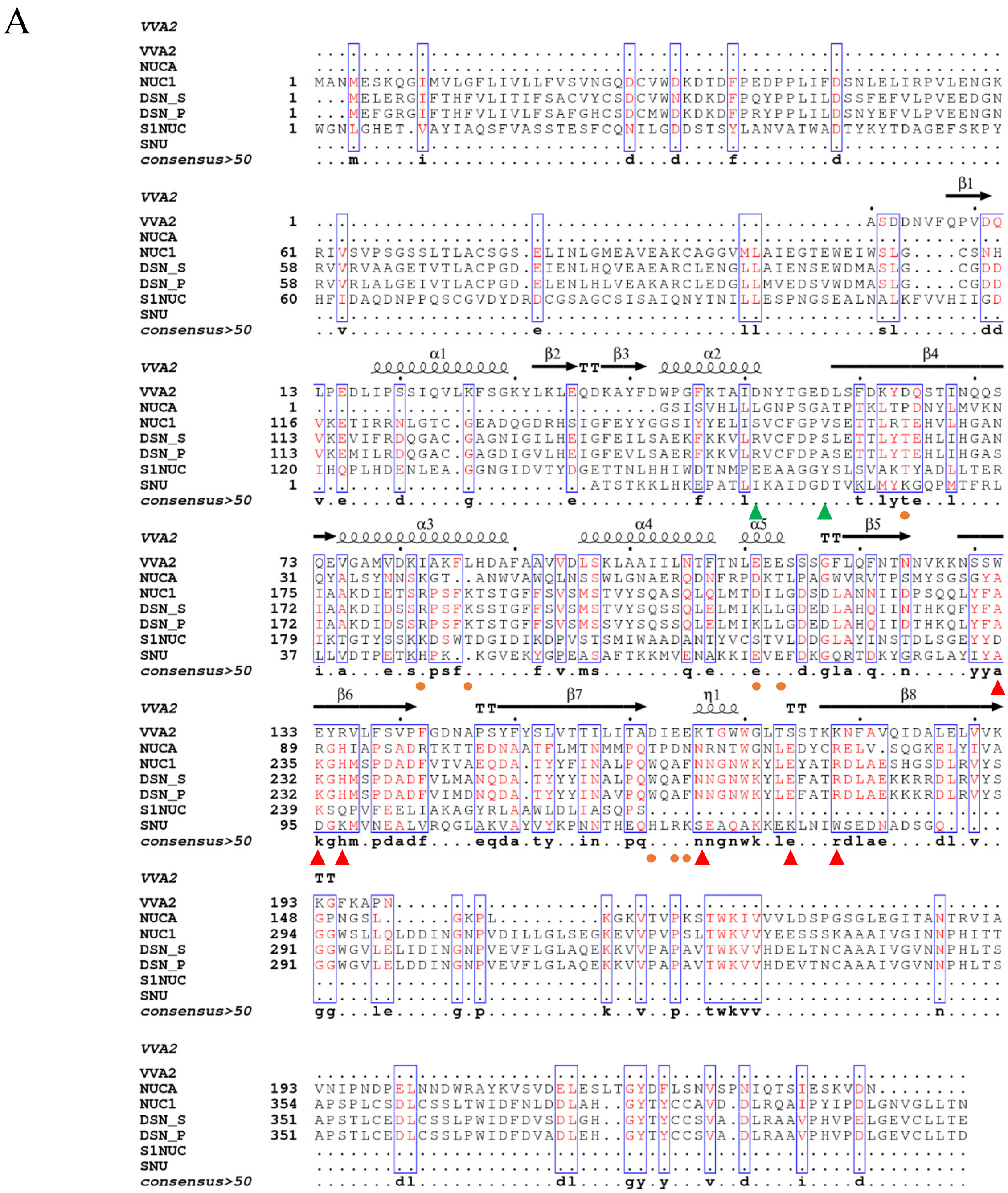
A
VVA2
NUCA
NUC1
DSN_S
DSN_P
S1NUC
SNU
consensus>50
β1 β2 β3 β4 β5 β6 β7 β8
α1 α2 α3 α4 α5
η1
TT

B

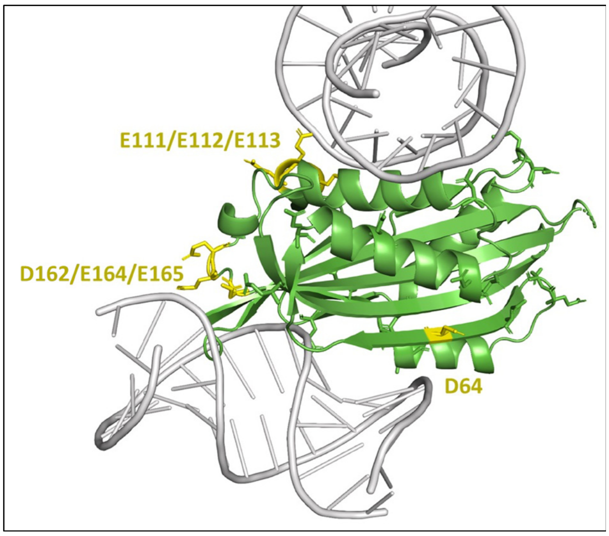

Figure S2. The multiple sequence alignment and structural comparison of VVA2 and other members of sugar non-specific nuclease. (**A**) The multiple sequence alignment and structural comparison of VVA2 and other members of sugar non-specific nuclease. The alignment was conducted as stated in Material and Methods. Related active sites were highlighted with red (for duplex-specific nucleases) and green (for non-specific nucleases). Amino acids of VVA2 involved in the mutagenesis study in this work were marked with orange dots. SNU: nuclease from *Staphylococcus aureus* (PDB code: 1STN_1); NUCA: nuclease A, nuclease from *Anabaena* sp. (PDB code: 2O3B_1); VVA2: Volvatoxin A2 (PDB code: 1VGF_A); S1NUC: S1 nuclease, nuclease from *Aspergillus oryzae* RIB40 (PDB code: 5FBF_1); NUC1: duplex-specific nuclease from *Paralithodes camtschaticus* (GenBank: AF520591.1). DSN1: duplex-specific nuclease from *Scylla paramamosain* (GenBank: AFP19103.1); DSN2: duplex-specific nuclease from *Portunus pelagicus* (GenBank: ADZ58187.1); (**B**) Molecular docking of 16-mer DNA (PDB code: 1CDW) on VVA2 (PDB code: 1PP0_A), as stated in Material and Methods. VVA2 is shown in the green cartoon; 16-mer DNA is shown in grey90. All the Glu and Arg are shown as sticks. Amino acids around the two clefts are shown in yellow. The codes of selected residues for further mutagenesis studies are marked in yellow and in bold.

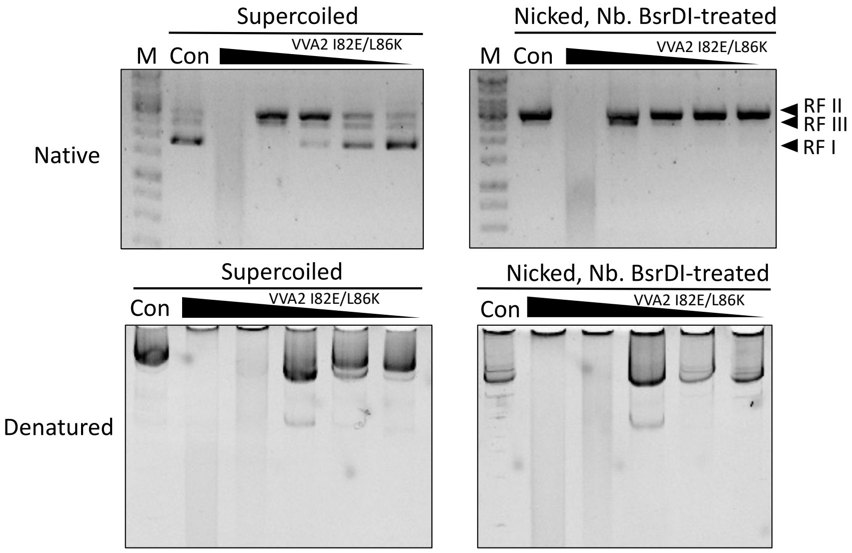

Figure S3. The comparison of nuclease activity of Re-VVA2 I82E/L86K on supercoiled and nicked dsDNA. Re-VVA2 I82E-L86K (16790 nM - 134.32 nM, 5-fold dilution) was used to cleave both supercoiled and nicked (by Nb. BsrDI) pUC19 (22.26 nM) at 37°C for 1.5 h. 1% agarose gel (upper) and 8 M Urea/ 6% acrylamide gel (lower) was used to analyze the sample after ethanol precipitation. M: GeneRuler 1 kb DNA Ladder. CON: control group, supercoiled/nicked pUC19 incubated with other reagents except for VVA2.

Table S1. The cleavage sites of Re-VVA2-I82E/L86K on pUC19 and pET28a.

No.	Substrate	Order	Sequence information	Location on plasmid
1	pUC19	Top: 5′-3'<br>Bottom: 3′-5'	TGGAT GGAGG//CGGAT AAAGT<br>ACCTA CCTCC//GCCTA TTTCA	1915
2	pUC19	Top: 5′-3'<br>Bottom: 3′-5'	GTTTG CCGGA//TCAAG AGCTA<br>CAAAC GGCCT//AGTTG TCGAT	2395
3	pUC19	Top: 5′-3'<br>Bottom: 3′-5'	GGTGG GTTAC//ATCGA ACTGG<br>CCACC CAATG//TAGCT TGACC	1415
4	pUC19	Top: 5′-3'<br>Bottom: 3′-5'	TACGG ATGGC//ATGAC AGTAA<br>ATGCC TACCG//TACTG TCATT	1625
5	pUC19	Top: 5′-3'<br>Bottom: 3′-5'	AAAGT TGCAG//GACCA CTTCT<br>TTTCA ACGTC//ATGGT GAAGA	2135
6	pUC19	Top: 5′-3'<br>Bottom: 3′-5'	ACTGA TTAAG//CATTG GTAAC<br>TGACT AATTC//GTAAC CATTG	1930
7	pUC19	Top: 5′-3'<br>Bottom: 3′-5'	GATGC CGCAT//AGTTA AGCCA<br>CTACG GCGTA//TCAAT TCGGT	937
8	pUC19	Top: 5′-3'<br>Bottom: 3′-5'	CCAGT GGCGA//TAAGT CGTGT<br>GGTCA CCGCT//ATTCA GCACA	2566
9	pUC19	Top: 5′-3'<br>Bottom: 3′-5'	GGCAT GACAG//TAAGA GAATT<br>CCGTA CTGTC//ATTCT CTTAA	2120
10	pUC19	Top: 5′-3'<br>Bottom: 3′-5'	TGAGA TAGGT//GCCTC ACTGA<br>ACTCT ATCCA//CGGAG TGACT	1633
11	pUC19	Top: 5′-3'<br>Bottom: 3′-5'	CATAC CTCGC//TCTGC TAATC<br>GTATG GAGCG//AGACG ATTAG	2527
12	pUC19	Top: 5′-3'<br>Bottom: 3′-5'	TACAC TTTAT//GCTTC CGGCT<br>ATGAG AAATA//CGAAG GCCGA	553
13	pUC19	Top: 5′-3'<br>Bottom: 3′-5'	GTTCT TCTAG//TGTAG CCGTA<br>CAAGA AGATC//ACATC GGCAT	2469
14	pUC19	Top: 5′-3'<br>Bottom: 3′-5'	CCGTA TTGAC//GCCGG GCAAG<br>GGCAT AACTG//CGGCC CGTTC	1533
15	pUC19	Top: 5′-3'<br>Bottom: 3′-5'	CGGAT GGCAT//GACAG TAAGA<br>GCCTA CCGTA//CTGTA ATTCT	1627
16	pUC19	Top: 5′-3'<br>Bottom: 3′-5'	AAAGC GCCAC//GCTTC CCGAA<br>TTTCG CGGTG//CGAAG GGCTT	37
17	pUC19	Top: 5′-3'<br>Bottom: 3′-5'	TCCGG CTCGT//ATGTT GTGTG<br>AGGCC GAGCA//TACAA CACAC	565
18	pET28a	Top: 5′-3'<br>Bottom: 3′-5'	TTTGA CGAGG//GGAAA TTAAT<br>AAACT GCTCC// CCTTT AATTA	4620
19	pET28a	Top: 5′-3'<br>Bottom: 3′-5'	GGACT GTTGG//GCGCC ATCTC<br>CCTGA CAACC//CGCGG TAGAG	581
20	pET28a	Top: 5′-3'<br>Bottom: 3′-5'	GGCAT CAGTG//ACCAA ACAGG<br>CCGTA GTCAC// TGGTT TGTCC	2667

21	pET28a	Top: 5′-3'<br>Bottom: 3′-5'	CGCTG CGCTC//GGTCG TTCGG<br>GCGAC GCGAG//CCAGC AAGCC	3137
22	pET28a	Top: 5′-3'<br>Bottom: 3′-5'	GCGCT GGCAG//TGTTC CTGCG<br>CGCGA CCGTC//ACAAG GACGC	4371
23	pET28a	Top: 5′-3'<br>Bottom: 3′-5'	GAGTT TGGAA//CAAGA GTCCA<br>CTCAA ACCTT//GTTCT CAGGT	5050
24	pET28a	Top: 5′-3'<br>Bottom: 3′-5'	GCACC GGGAT//CTCGA CCGAT<br>CGTGG CCCTA//GAGCT GGCTA	1902

Table S2. Primers used for cloning and site-direct mutagenesis[1] studies of VVA2.

Variants	Restriction enzyme	Forward primer (5′-3′)	Reverse primer (5′-3′)
VVA2-I82E/L86K	BamH I & Hind III	AATTCCGGATCCATGGCAA GTGATGAT	GGAATTAAGCTTTTAAAGCTT ATTCGG
VVA2- D64A/I82E/L86K	-	GATAAATATGCTCAGAGCA CC	GGTGCTCTGAGCATATTTATC
VVA2- E111A/E112A/E113A/ I82E/L86K	-	ACCAACCTGGCAGCAGCAAG CAGCAGC	GCTGCTGCTTGCTGCTGCCAG GTTGGT
VVA2- D162A/E164A/E165A/ I82E/L86K	-	ATTACCGCAGCTATTGCAGC AAAAACCGGT	ACCGGTTTTTGCTGCAATAGC TGCGGTAAT
VVA2- E111A/I82E/L86K	-	ACCAA CTGGCAGAAGAAAGCAGCA GC	GCTGCTGCTTTCTTCTGCCAGG TTGGT
VVA2- E112A/I82E/L86K	-	ACCAACCTGGAAGCAGAAA GCAGCAGC	GCTGCTGCTTTCTGCTTCCAGG TTGGT
VVA2- E111A/E112A/I82E/L86 K	-	ACCAACCTGGCAGCAGAAA GCAGCAGC	GCTGCTGCTTTCTGCTGCCAG GTTGGT

[1] Overlapping PCR method was used for site-direct mutagenesis
